# Clinicopathological features and prognosis of patients with HER2-low breast cancer

**DOI:** 10.1186/s12885-023-11421-0

**Published:** 2023-09-28

**Authors:** Xin Yang, Yao Li, Xu lu, Xiaotian Ren, Bin Hua

**Affiliations:** Breast Center, Department of Thyroid–Breast–Hernia Surgery, Department of General Surgery, Beijing Hospital, National Center of Gerontology, Institute of Geriatric Medicine, Chinese Academy of Medical Sciences, Beijing, People’s Republic of China

**Keywords:** Breast cancer, HER2-low expression, Clinicopathological features, Prognosis

## Abstract

**Background:**

Low human epidermal growth factor receptor 2 (HER2) expression is an emerging concept in breast cancer that is defined as immunohistochemistry (IHC) 1 + or IHC 2 + and negative in situ hybridization (ISH) but has been poorly investigated. The aim of our study was to determine the frequency of low HER2 expression among HER2-negative breast cancers and compare the clinicopathological features and prognosis of HER2-low patients with those of HER2-zero patients.

**Methods:**

We collected the data of 684 patients with primary HER2-negative breast cancer who underwent surgery between January 2012 and September 2021 from our self-built database. Clinicopathological features, recurrence-free interval (RFI) and breast cancer-specific survival (BCSS) were compared between HER2-low and HER2-zero (IHC 0) patients.

**Results:**

Among the 684 patients, 512 (74.9%) patients had low HER2 expression, and 172 (25.1%) patients had zero HER2 expression. The average age was 57.7 ± 12.6 years, 472 (69.0%) patients were aged < 65 years, and 212 (31.0%) patients were aged ≥ 65 years. Compared to HER2-zero tumors, HER2-low tumors had a higher proportion of hormone receptor (HR)-positive cases (89.6% vs. 75.6%, *P* < 0.001) and a lower rate of histological grade III cases (29.4% vs. 38.8%, *P* < 0.001). Kaplan–Meier analysis showed that low HER2 expression was associated with prolonged RFI in breast cancer patients, especially in HR + breast cancer patients (*P* = 0.028) and < 65-year-old breast cancer patients (*P* = 0.000). Multivariate Cox regression analysis showed that low HER2 expression was a low-risk factor for RFI (HR: 0.531, 95% CI: 0.292–0.967, P = 0.038) but had no influence on BCSS (*P* = 0.272).

**Conclusions:**

HER2-low tumors had a higher proportion of HR positivity and a lower rate of histological grade III than HER2-zero tumors. Low HER2 expression seemed to be a protective factor for RFI, especially in patients with HR + and those younger than 65 years.

## Introduction

Breast cancer is the most commonly diagnosed malignant tumor in the world [1, 2]. Human epidermal growth factor receptor 2 (HER2) is an important biomarker of poor prognosis and a therapeutic target for anti-HER2 agents when overexpressed in breast cancer [3–5]. According to current recommendations, HER2 positivity is diagnosed when the immunohistochemistry (IHC) result is 3 + or 2 + with ERBB2 gene amplification detected by in situ hybridization (ISH). Tumors with IHC 0, IHC 1 + and IHC 2 + with negative ISH are classified as HER2 negative [6]. However, some HER2-negative tumors (HER2 1 + and HER2 2+) can also express certain levels of HER2 protein on the cell surface when detected by IHC, which are now called HER2-low tumors.

HER2-targeted therapy has been proven to significantly improve the prognosis of patients with HER2-positive breast cancer [7], while the addition of trastuzumab to adjuvant chemotherapy did not improve the prognosis of patients with HER2-low breast cancer, as shown in NSABP B-47 [8]. However, novel antibody‒drug conjugates (ADCs), such as trastuzumab deruxtecan and trastuzumab duocarmazine, have shown antitumor activity in HER2-low advanced/metastatic tumors [9–11]. For example, in DESTINY-Breast04, trastuzumab deruxtecan (an ADC composed of a humanized anti-HER2 monoclonal antibody and a topoisomerase I inhibitor payload) significantly improved progression-free survival (PFS) and overall survival (OS) compared with the physician’s choice of chemotherapy in patients with HER2-low metastatic breast cancer. These findings suggest that low expression of HER2 protein on the cell surface could be a therapeutic target for ADCs.

A few studies have investigated the clinicopathological features and prognostic value of low HER2 expression. Some studies showed that HER2-low tumors had larger tumor sizes and more nodal involvement, and low HER2 expression was associated with poor prognosis [12, 13]. Other studies found different results that HER2-low tumors had smaller tumor sizes and lower histological grades, and HER2-low patients had better outcomes than HER2-zero patients [14]. To deepen the understanding of the clinicopathological features and prognosis of patients with HER2-low breast cancer, we retrospectively analyzed the data of our hospital from January 2012 to September 2021, comparing the differences between HER2-low and HER2-zero patients. The results are reported herein, including those of stratified analyses based on hormone receptor (HR) status and age.

## Methods

### Patients

This study is a single-center retrospective research, early breast cancer patients who underwent surgery between January 2012 and September 2021 were identified from our self-built database. The database was initiated in January 2012, with follow-up conducted every six months to gather patient survival information. Information such as age, T stage, N stage, HR, HER2, Ki67, histological grade and follow-up were included in the database. Patients who met the following criteria were included: pathologically confirmed primary tumor, HER2 negative (IHC 0, 1 + and 2+/ISH negative), and follow-up longer than 3 months. Patients with HER2 positivity and IHC 2 + but no ISH detection were excluded. The study protocol was approved by the Ethics Committee of Beijing Hospital on the basis of the Declaration of Helsink (IRB Number in Ethical approval: 2022BJYYEC-049-01), and written informed consent was obtained from the patients or their legal guardians.

### HR and HER2 classification

Tumor samples with > 1% of tumor nuclei positive for estrogen receptor (ER) or progesterone receptor (PR) were considered ER/PR positive. HR positivity was defined as ER and/or PR positivity [15].

The HER2 level was assessed by IHC and ISH according to the most recent version of the American Society of Clinical Oncology/College of American Pathologists Clinical Practice (ASCO/CAP) guidelines at the time of surgery [6, 16]. HER2-low was defined as IHC 1 + and IHC 2 + with negative ISH. HER2-zero was defined as IHC 0.

### Follow-up and statistical analysis

Postoperative follow-up was performed every 3–6 months in the first 3 years and annually thereafter, and the deadline was December 31, 2021. The recurrence-free interval (RFI) was defined as the time from surgery to local-regional recurrence or distant metastasis. Breast cancer-specific survival (BCSS) was defined as the time from surgery to death from breast cancer.

All statistical analyses were performed using SPSS version 21.0 (SPSS Inc., Chicago, IL, USA). The chi-square test and independent t test were used to compare the clinicopathological features. The Kaplan‒Meier method was used to generate survival curves, and the log-rank test was used to compare the differences in RFI and BCSS. Then, we stratified these data by HR status (HR + vs. HR-) and age (< 65 vs. ≥ 65) and compared the differences between these subgroups. Univariate Cox proportional hazards regression analysis was used to assess the association of each factor with prognosis, and multivariate analysis was used to evaluate the prognostic significance. All statistical tests were two-sided, and *P* < 0.05 was considered significant.

## Results

### Patients and clinicopathological features

A total of 1452 early breast cancer patients underwent surgery between January 2012 and September 2021. After excluding patients with HER2 positivity and IHC 2 + without ISH detection, 684 patients were included in this study. The average age was 57.7 ± 12.6 years (ranging from 26 to 89), 472 (69.0%) patients were aged < 65 years, and 212 (31.0%) patients were aged ≥ 65 years. We identified 512 (74.9%) HER2-low patients (294 patients with HER2 1+, 218 patients with HER2 2 + and ISH-) and 172 (25.1%) HER2-zero patients. According to HR status, 95 (13.9%) patients had HR-negative tumors, and 589 (86.1%) patients had HR-positive tumors.

Compared with HER2-zero tumors, HER2-low tumors had a higher proportion of HR + tumors (89.6% vs. 75.6%, *P* < 0.001) and a lower proportion of grade III tumors (29.4% vs. 38.8%, *P* < 0.001). There were no significant differences in age, tumor size, lymph node status, Ki67, vascular invasion or perineural invasion between the HER2-low group and the HER2-zero group (Table 1).

**Table 1:** Clinicopathological features by HER2 expression

Characteristics	No. of patients	HER2-zero, n(%)	HER2-low, n(%)	^a^*p* value
Age				
Mean age ± St.deviation		58.2 ± 13.95	57.5 ± 12.35	0.560
≥ 65	212	59(34.3)	153(29.9)	0.141
<65	472	113(65.7)	359(70.1)	
T stage				
T1	365	81(47.1)	284(55.5)	0.180
T2	297	84(48.8)	213(41.6)	
T3	22	7(4.1)	15(2.9)	
N stage				
N0	439	111(64.5)	328(64.1)	0.172
N1	141	40(23.3)	101(19.7)	
N2	61	16(9.3)	45(8.8)	
N3	43	5(2.9)	38(7.4)	
HR status				
Negative	95	42(24.4)	53(10.4)	**0.000**
Positive	589	130(75.6)	459(89.6)	
Ki67				
≤ 14	236	53(31.8)	183(36.0)	0.350
> 14	440	114(68.3)	326(64.0)	
Missing	8	5	3	
Vascular invasion				
Negative	606	156(90.7)	450(87.9)	0.405
Positive	78	16(9.3)	62(12.1)	
Perineural invasion				
Negative	624	162(94.2)	462(90.2)	0.122
Positive	60	10(5.8)	50(9.8)	
Histological grade				
I	95	22(13.3)	73(14.5)	**0.000**
II	407	79(47.9)	328(65.1)	
III	167	64(38.8)	103(20.4)	
Missing	15	7	8	
Chemotherapy				
Yes	481	122(70.9)	359(70.1)	0.840
No	203	50(29.1)	153(29.9)	

^a^*p* values were derived from chi-square test

**Bold figure note:** this variable is statistically significant

A total of 480 patients received either preoperative or postoperative chemotherapy. Among them, there were 122 cases (preoperative chemotherapy: 10 cases) in the HER2-zero group and 358 cases (preoperative chemotherapy: 31 cases) in the HER2-low group. There were no statistically significant differences between the two groups (70.9% vs. 69.9%, *P* = 0.803).

### Low HER2 expression seemed to be a protective factor for RFI

**Fig. 1:**
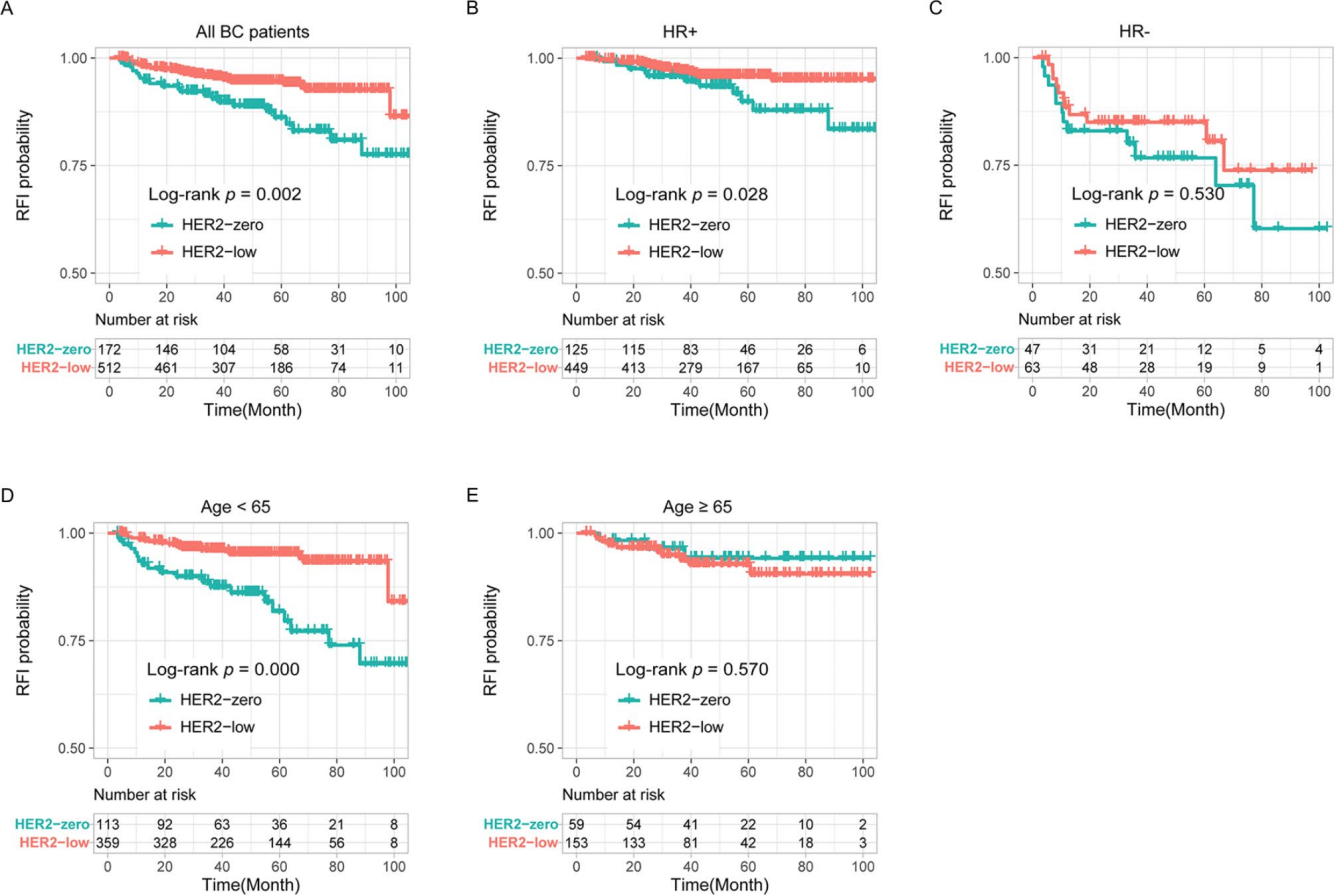
Low HER2 expression was correlated with longer RFI in the overall patient cohort **(A)**, HR + patients **(B)** and patients aged < 65 years **(D)** but not in HR- patients **(C)** and patients aged ≥ 65 years **(E)**. Abbreviation: HR, hormone receptor

Over a median follow-up of 52.7 months (ranging from 3 months to 107 months), a total of 51 RFI events were recorded, of which 10 patients had local-regional recurrence and 41 patients had distant metastasis. There were 23 RFI events in the HER2-zero group and 28 RFI events in the HER2-low group. Then, Kaplan–Meier curves of RFI showed that patients in the HER2-low group had a longer RFI than patients in the HER2-zero group (*P* = 0.002, Fig. 1A). A similar trend was found in HR + patients (*P* = 0.028, Fig. 1B) but not in HR- patients (*P* = 0.530, Fig. 1C). Moreover, analysis of RFI according to age showed that HER2-low expression was associated with a longer RFI in patients aged < 65 years (*P* = 0.000, Fig. 1D), and no significant difference was observed between the HER2-low and HER2-zero groups in patients aged ≥ 65 years (*P* = 0.570, Fig. 1E).

Univariate and multivariate Cox proportional hazard models were used to identify prognostic factors affecting RFI. Low HER2 expression was independently associated with a longer RFI (HR: 0.531, 95% CI: 0.292–0.967, *P* = 0.038). Other factors included HR status, T stage and N stage (Table 2).

**Table 2:** Univariate and multivariate cox regression analyses of Clinicopathological features to explore the RFI.

Variables	Univariate			Multivariate		
	^a^HR	95%CI	^b^*p* value	HR	95%CI	^b^ *p*
HER2(zero vs. low)	0.427	0.246–0.742	**0.003**	0.531	0.292–0.967	**0.038**
^c^HR(negative vs. positive)	0.193	0.110–0.338	**0.000**	0.216	0.117–0.397	**0.000**
Ki67(< 14% vs. ≥ 14%)	2.281	1.143–4.555	**0.019**	1.293	0.615–2.719	0.498
T stage(T1 vs. T2/3)	2.631	1.456–4.755	**0.001**	2.161	1.302–3.587	**0.003**
N stage(N0 vs. N1/2/3)	4.722	2.582–8.637	**0.000**	2.243	1.728–2.911	**0.000**
perineural invasion(negative vs. positive)	0.837	0.260–2.698	0.766			
vascular invasion(negative vs. positive)	1.752	0.779–3.940	0.175			
Grade(I/II vs. III)	1.912	1.069–3.421	**0.029**	2.500	0.329–18.985	0.376

**Abbreviations:** ^a^HR, hazard ratio; CI, confidence interval; ^*b*^*p* values were derived from chi-square test; ^c^HR, hormone receptor

**Bold figure note:** this variable is statistically significant

### Low HER2 expression was not significantly associated with BCSS

**Fig. 2:**
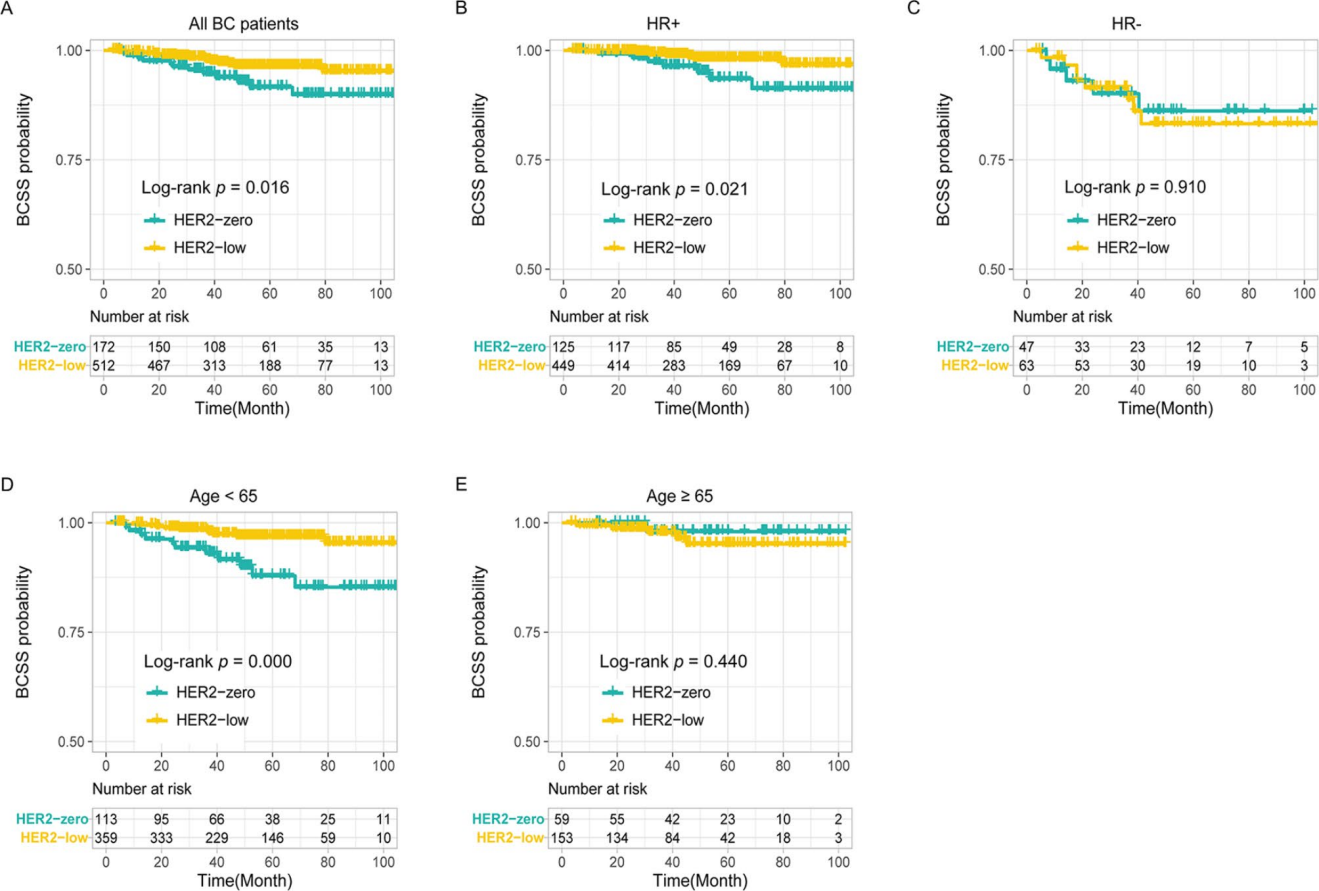
Low HER2 expression was correlated with longer BCSS in the overall patient cohort **(A)**, HR + patients **(B)** and patients aged < 65 years **(D)** but not in HR- patients **(C)** and patients aged ≥ 65 years **(E)**. Abbreviation: HR, hormone receptor

Thirty-five patients died during the follow-up: 27 patients died of breast cancer, and 8 patients died of other diseases. Twelve BCSS events occurred in the HER2-zero group, and 15 BCSS events occurred in the HER2-low group. Patients in the HER2-low group had a longer BCSS than those in the HER2-zero group (*P* = 0.016, Fig. 2A). Similar to the RFI analysis, this trend was found in HR + patients (*P* = 0.021, Fig. 2B) and patients aged < 65 years (*P* = 0.000, Fig. 2D) but not in HR- patients (*P* = 0.910, Fig. 2C) or patients aged ≥ 65 years (*P* = 0.440, Fig. 2E). However, after univariate and multivariate analyses, no statistical association was found between HER2 expression and BCSS (Table 3).

**Table 3:** Univariate and multivariate cox regression analyses of Clinicopathological features to explore the BCSS.

Variables	Univariate			Multivariate		
	^a^HR	95%CI	^b^*p* value	HR	95%CI	^b^*p* value
HER2(zero vs. low)	0.454	0.211–0.973	**0.043**	0.711	0.277–1.377	0.272
^c^HR(negative vs. positive)	0.215	0.097–0.473	**0.000**	0.252	0.100-0.632	**0.003**
Ki67(< 14% vs. ≥ 14%)	3.260	1.126–9.437	**0.029**	2.566	0.730–9.024	0.142
T stage(T1 vs. T2/3)	2.639	1.147–6.071	**0.022**	1.812	0.769–4.270	0.174
N stage(N0 vs. N1/2/3)	5.001	2.102–11.899	**0.000**	3.944	1.623–9.586	**0.002**
perineural invasion(negative vs. positive)	0.557	0.075–4.120	0.566			
vascular invasion(negative vs. positive)	2.050	0.695–6.043	0.193			
Grade(I/II vs. III)	2.595	1.178–5.718	**0.018**	1.041	0.407–2.662	0.933

**Abbreviations:** ^a^HR, hazard ratio; CI, confidence interval; ^*b*^*p* values were derived from chi-square test; ^c^HR, hormone receptor

**Bold figure note:** this variable is statistically significant

### The relationship between age and clinicopathological features as well as prognosis

As indicated in Table 4, patients aged < 65 years exhibited a higher incidence of lymph node positivity (40.3% vs. 26.2%, *P* = 0.000) and a greater prevalence of high Ki67 expression (67.7% vs. 59.3%, *P* = 0.036) compared to patients aged ≥ 65 years. Notably, the percentage of patients age < 65 years who received chemotherapy was significantly higher than that of patients aged ≥ 65 (81.1% vs. 46.2%, *P* = 0.000) regardless of the pathological subtype (Table 5).

**Table 4:** Clinicopathological features by age

Characteristics	No. of patients	Age < 65, n(%)	Age ≥ 65, n(%)	^a^*p* value
HER2				
HER2-zero	172	113(23.9)	59(27.8)	0.278
HER2-low	512	359(76.1)	153(72.2)	
HR				
HR+	574	393(83.3)	181(85.4)	0.486
h-	110	79(16.7)	31(14.7)	
T stage				0.852
T1	365	253(53.6)	112(52.8)	
T2/3	319	219(46.3)	100(47.2)	
N stage				
N0	437	282(59.7)	155(73.8)	**0.000**
N1/2/3	245	190(40.3)	55(26.2)	
Ki67				
≤ 14	236	151(32.3)	85(40.7)	**0.036**
> 14	440	316(67.7)	124(59.3)	
Missing	8	5	3	
Histological grade				
I/II	505	339(73.7)	166(79.4)	0.110
III	164	121(26.3)	43(20.6)	
missing	15	12	3	
Chemotherapy				
Yes	481	383(81.1)	98(46.2)	**0.000**
No	203	89(18.9)	114(53.8)	

^a^*p* values were derived from chi-square test

**Bold figure note:** this variable is statistically significant

**Table 5:** The administration of chemotherapy among patients with different pathological subtypes

Subtype	Age < 65, n(%)	Age ≥ 65, n(%)	^a^*p* value
Luminal	307(78.1)	23(41.4)	**0.000**
TNBC	76(96.2)	23(74.2)	**0.000**

^a^*p* values were derived from chi-square test

**Bold figure note:** this variable is statistically significant

In patients age < 65 years, there was no significant difference in the proportion of chemotherapy administration among HER2-low and HER2-zero patients regardless of the pathological subtype (Table 6). After univariate and multivariate analyses, those with HER2-low tumors have a longer RFI (HR: 0.430, 95% CI: 0.216–0.856, *P* = 0.016) and BCSS (HR: 0.384, 95% CI: 0.158–0.931, *P* = 0.0034). Chemotherapy had no impact on the prognosis of patients (*P* > 0.05).

**Table 6:** The administration of chemotherapy among patients with different pathological subtypes in patients age < 65 years

Subtype	HER2-low, n(%)	HER2-zero, n(%)	^a^*p* value
Luminal	251(79.4)	55(72.4)	0.182
TNBC	41(95.3)	35(97.2)	0.664

^a^*p* values were derived from chi-square test

**Bold figure note:** this variable is statistically significant

## Discussion

With the application of ADCs in metastatic HER2-low breast cancers, HER2-low expression has received increasing attention [17, 18]. However, the clinicopathological features and prognosis of HER2-low tumors remain poorly investigated, especially in Chinese patients. In this retrospective study, we identified 684 HER2-negative breast cancers to detect differences between HER2-zero and HER2-low tumors. The results showed that HER2-low tumors had a higher proportion of HR positivity and a lower proportion of histological grade 3. Moreover, low HER2 expression seemed to be a protective factor for RFI, especially in HR + patients and patients aged < 65 years.

A few studies have focused on low HER2 expression in HER2-negative breast cancer. Schettini et al. evaluated 3689 HER2-negative cases from the cBio Cancer Genomics Portal, and 59.4% of patients had low HER2 expression [12]. A similar proportion (61%) was found by Agostinetto et al., who evaluated 804 cases from TCGA [19]. Interestingly, the proportion of HER2-low patients was higher in Asian patients. In a retrospective study of 4918 HER2-negative patients from Japan, 79.1% of patients had HER2-low tumors [20]. In Chinese patients, a retrospective study of 12,467 patients reported that the proportion of HER2-low tumors was 72.6% [21], which was consistent with our results (74.9%). However, clinicopathological features and prognosis were not further explored in this study. The differences in the HER2-low proportion may be due to racial differences, disease staging, and quality control of HER2 detection.

Furthermore, we found that HER2-low tumors had a higher proportion of HR positivity than HER2-zero tumors (89.6% vs. 75.6%, *P* < 0.01), which was consistent with the findings of previous studies (90.2% in Japanese populations and 88.2% in cases from the cBio Cancer Genomics Portal) [12, 20]. These differences may stem from variations in gene expression according to Schettini et al.‘s research [12]. In their study, compared to HER2-zero breast cancer, the expression of luminal-related genes such as BCL2 and FOXA1 was upregulated in HER2-low breast cancer [22, 23]. Conversely, the expression of basal-like related genes such as KRT14 and FOXC1 was downregulated, resulting in a higher proportion of HR + tumors in HER2-low cases [24, 25]. Other clinicopathological features varied across different studies. Horisawa et al. found that HER2-low tumors have a smaller tumor size and lower proportion of histological grade 3 [20], and similar results were found in triple-negative breast cancer (TNBC) by Jacot et al. [26]. Schettini et al. found worse T stages, N stages and histological grades in HER2-low tumors than in HER2-zero tumors [12]. We observed a lower proportion of histological grade 3 in low HER2 expression patients. The reasons for these differences are unclear, and more studies are needed.

In regard to prognosis, previous studies have shown different results. A retrospective study by Yiqun Li et al. involving 1433 patients with metastatic breast cancer reported that patients with low HER2 expression survived longer in the overall population and HR + subgroup [14]. Another study by Dehgani et al. in TNBC obtained a similar result: patients with HER2 2 + had a lower rate of recurrence and longer overall survival (OS) [27]. In addition, other studies found no statistically significant difference in OS between patients with HER2-low and HER2-zero tumors [12, 19]. Conversely, a retrospective study including 91 node-positive patients found that low HER2 expression was associated with shorter disease-specific survival (DFS) and OS, and the correlation was more significant in HR + patients [28]. In another study of 5907 patients, moderate HER2 expression (HER2 2+) was also considered an adverse factor for DFS [13]. The different results of previous studies may be caused by several reasons. First, the inclusion criteria varied in different studies; some focused on TNBC, and some focused on early-stage or advanced breast cancer. Second, as an important prognostic factor, therapeutic regimens were not mentioned in most studies. Third and most importantly, breast cancer with low HER2 expression may be a highly heterogeneous disease, and more efforts are needed to define HER2 levels.

Age is an important factor affecting the prognosis of breast cancer but is poorly investigated in HER2-low patients. In our study, there was no significant difference in the proportion of HER2 statuses between patients aged < 65 years and ≥ 65 years. In the patients age < 65 years, there was a similar proportion of patients receiving chemotherapy between the HER2-low and HER2-zero groups both in the Luminal and TNBC subtypes. The Cox analysis results indicated that patients with HER2-low tumors exhibited longer RFI and BCSS, while chemotherapy had no discernible impact on prognosis. This is an intriguing finding, and we did not find similar studies focusing on age. However, given the limitations of our sample size, further validation with additional data may be warranted to confirm this result.

Our study has several limitations. First, although we have relatively complete clinicopathological and follow-up data in our database, this was a single-center retrospective study. Second, different criteria for HER2 evaluation were used due to the updating of the ASCO guidelines. Third, some patients with HER2 2 + did not undergo ISH detection and were not included in this analysis. However, we provided data from Chinese patients with HER2-low early breast cancer and performed analyses stratified by HR status and age.

## Conclusions

In summary, our study indicates that HER2-low and HER2-zero breast cancer have different clinicopathological features and prognoses. Furthermore, low HER2 expression seems to be a protective factor for RFI. Further investigations are needed to deepen the understanding of HER2-low breast cancer.

## Data Availability

All data generated or analyzed during this study are included in this published article.
